# Growing Strong and Healthy with Mister Bone: An Educational Program to Have Strong Bones Later in Life

**DOI:** 10.3390/nu7125510

**Published:** 2015-12-02

**Authors:** Barbara Pampaloni, Luisella Cianferotti, Giorgio Gronchi, Elisa Bartolini, Sergio Fabbri, Annalisa Tanini, Maria Luisa Brandi

**Affiliations:** Metabolic Bone Diseases Unit, Department of Surgery and Translational Medicine, University of Florence, Viale Pieraccini 6, Florence 50139, Italy; barbara.pampaloni@unifi.it (B.P.); luisella.cianferotti@unifi.it (L.C.); giorgio.gronchi@gmail.com (G.G.); elisabartolini@yahoo.it (E.B.); sergio.fabbri@unifi.it (S.F.); annalisa.tanini@unifi.it (A.T.)

**Keywords:** health, nutrition, education, children, school, osteoporosis, prevention, calcium intake, rickets

## Abstract

Optimal peak bone mass and bone health later in life are favored by a sufficient calcium intake in infancy, childhood and adolescence. The purpose of this study was to test a new educational program created to monitor and to improve calcium and vitamin D intake in children. Nutritional habits in children were evaluated through a food frequency questionnaire (FFQ) to assess the intake of calcium, vitamin D, dairy products, and total caloric energy at baseline and after seven months of exposure to a unique educational program applied between November 2013 and May 2014 in 176 schoolchildren (48% male, 52% female) attending the fourth and fifth grades of two selected primary schools in Florence, Italy. A significant increase of calcium (from 870 ± 190 to 1100 ± 200 mg/day, *p* < 0.05), and vitamin D (from 3.6 ± 1.53 to 4.1 ± 2 µg/day) intake in children was documented after the educational program. The amount of specific foods important for bone health consumed, such as milk and vegetables, increased significantly, both in male and female children (*p* < 0.05). The proposed educational program appears to be effective in modifying calcium intake in children, with a significant increase in the consumption of dairy products and vegetables, but without a significant change in the total caloric intake.

## 1. Introduction

Osteoporosis is a chronic disease characterized by low bone mass and deterioration of bone microarchitecture, leading to skeletal fragility and increased fracture risk [1]. Osteoporosis is a global public health burden affecting more than 200 million people worldwide and causing more than nine million fragility fractures every year (data from International Osteoporosis Foundation) [2]. Hip fractures, a major cause of excess morbidity and mortality, have high comparable rates in USA and Central/Southern European countries (150–250/100,000 for both sexes combined) and even higher incidence in Northern European countries (>250/100,000 for both sexes combined) [3]. Bone mass increases steadily until age 20–30 years, and the first two decades of life are critical for peak bone mass (PBM) acquisition [4]. Optimal nutrition plays a critical role in the achievement of the genetically programmed PBM, with reduction in the risk of osteoporosis later in life [5,6].

Bone is a living tissue, and bone cells have the same need for nutrients as the rest of the body, not just protein and energy supply, but also micronutrients, such as minerals (e.g., calcium, phosphate and magnesium) and vitamins (e.g., vitamin D, vitamin B, and vitamin K) [1,7,8,9,10,11]. The relationship between calcium intake and bone mineral density (BMD) has been extensively assessed. Numerous studies have demonstrated that the increase in BMD in children and adolescents is proportional to calcium intake [12,13,14]. Many authors have observed that by optimizing calcium intake in children and adolescents, either through dairy products or supplements, BMD increases by 4%–8%, depending on the study or the skeletal site measured [15,16,17].

In a balanced Western-style diet, about 60% of dietary calcium comes from milk and dairy products, 20% from fresh vegetables, nuts and dried fruits, and the rest from drinking water or other discrete sources.

While the intake of calcium and bone micronutrients is often inadequate in childhood and adolescence even in Mediterranean countries [18,19,20,21,22], a better bone-oriented nutrition (higher intake of yogurt and dairy foods) is associated with greater intake of shortfall nutrients and lower body fat in large national surveys [23]. In the Italian diet, mean Ca daily intake is about 738 mg/day, and milk-and-dairy foods make the greatest contribution, so that about 59% of the total daily intake of Ca derives from this food group [24]. In Italy, the INRAN (*Istituto Nazionale di Ricerca per gli Alimenti e la Nutrizione*, Italian Research Institute for Foods and Nutrition) data report on the Food Consumption Survey 2005–2006 showed that children aged 10–17 years consume a mean of 170 g/day of milk, and 13.5 g/day of yogurt, which together represent 10%–12% of the total energy in the diet [25]. Recent reports show that calcium intake in Italian primary school-age children is far below the recommended adequate intake of 1100–1300 mg/day [26]. Indeed, a recent study in 773 Italian adolescents aged 11–13 years showed that the daily calcium intake (815 mg/day) is under the recommended quantity for this population [27].

Vitamin D is essential for the absorption of calcium and phosphate, and for enhancing bone mineralization, with sunlight exposure being the primary determinant of vitamin D status [28]. Indeed, vitamin D deficiency is characterized by inadequate mineralization of the skeleton, with consequent development of rickets and osteomalacia in children, which undermine bone accrual and PBM [29]. Conversely, an increase in vitamin D intake significantly raises bone mineral density in adolescents [30]. The prevalence of vitamin D deficiency among infants, children, and adolescents is between 12% and 24%, with vitamin D nutritional intake often being below the recommended daily intake [31]. In a recent study, up to 82% of adolescents were found to have hypovitaminosis D (e.g., 25(OH) vitamin D below 75 nmol/L) [32].

These data point to the need of developing nutritional education programs for children in order to improve the dietary intake of calcium, vitamin D, protein and vegetables, with the goal of achieving the optimal PBM necessary to prevent osteoporosis and consequent fragility fractures.

Studying the eating behavior of children is certainly more difficult than assessing dietary patterns of adults, but it is a focal point for the primary prevention of chronic diseases.

In recent years, several studies have been conducted with the aim of improving eating patterns and lifestyle in school-age children, in order to prevent the onset of many chronic diseases later in life. However, many of these studies are used to hinder overweight and obesity, not specifically to improve calcium and vitamin D intakes, and are often limited to solutions in the school environment, not influencing nutrition at home [33,34,35,36,37].

The purpose of this pilot study was to investigate the effectiveness of a nutritional education program, specifically realized to improve calcium and vitamin D intake, in a reference group of healthy Italian primary schoolchildren (aged 9–11). The key feature of our study consisted in the use of new communication tools, able to convey the message of correct nutrition for healthy bones in a unique, simple and funny/gamified way, able to engage and empower the children.

## 2. Experimental Section

### 2.1. Study Group

In November 2013, a group of 176 schoolchildren, 48% male and 52% female, aged 9–10 years, were recruited from two selected primary schools in Florence. This age range was selected because it is critical for the achievement of optimal PBM necessary to prevent the onset of osteoporosis. Moreover, at this age children are perfectly capable of cooperating in the collection of nutritional data and are able to take advantage of computer-based resources and tools.

Participants were asked to complete a specific questionnaire in order to know their current eating habits. The children involved were not following any special diet, and were not taking any dietary supplements. Informed consent was obtained from their parents.

In May 2014, the second step of the project was carried out. At this stage, nutritional data was collected from 156 of the 176 subjects in order to evaluate the project efficacy. Specifically, each participant who was present at school the day of the second, unannounced, evaluation was asked to complete the questionnaire for a second time, in order to estimate possible changes in nutritional behavior as a result of the educational program.

The 7-month program took place during the school year, when food habits are presumed to be consistent.

Since data were collected in two different steps, *i.e.*, before and after the educational program, the study should be considered a “pre–post intervention study”, in which each interviewed subject is the control of his/herself.

The study was authorized by the local Internal Review Board of the University Hospital of Florence/University of Florence (Florence, Italy) in September 2013 (project identification code: 79.13). The research was conducted in accordance with the Declaration of Helsinki, and written informed consent for collection of personal data was obtained from all participants.

### 2.2. Instruments and Procedures for Nutritional Data Collection

A twenty-one item questionnaire (FFQ), specifically validated for the assessment of calcium and important nutrients for bone health in children [38], was administered to estimate the nutritional habits of the children, particularly to assess the intake of calcium, vitamin D, dairy products, and total energy. The questionnaire included the following foods: milk, yogurt, ice cream, cheese, pizza, and chocolate milk. Meat, fish, cereals and bread, vegetables, and fruits were also included.

Subjects were asked to report the frequency of consumption and portion size with the advice and supervision of two nutritionists. Color photographs to identify serving size were part of the questionnaire. Portions were classified in two sizes (B and C), displayed as a photograph for each item, corresponding to medium and large portions. The children could also indicate portions smaller than “B” or larger than “C”. Food frequency consumption of each item was evaluated using categories: daily, weekly (from once to six times a week), monthly (from one to three times a month), and never. Great attention was given by the nutritionists to explain how to complete the questionnaire, and the importance of a detailed and not manipulated response.

### 2.3. Educational Instruments and Intervention

The project was realized with the support of a novel instrument, specifically created with a publisher (Giunti Organizzazioni Speciali, Firenze) dedicated to the production of schoolbooks for children. The project messages and materials featured Mister Bone^®^, a bony little boy, who is the hero of a cartoon game series and several online games, all produced to attract children to the project, and also to involve the entire family.

The detailed steps of the nutritional education program are summarized as follow:

Step 1: Creation of a dedicated website [39] with the educational program, “online” games, and scientific material on bone health.

Step 2: Realization of the “Mister Bone Calendar”, an indispensable educational tool through which each child could check their physical activity and daily calcium intake.

Step 3: A classroom session conducted by nutritionists to teach children the importance of the adequate intake of calcium and other nutrients necessary for healthy bones.

Step 4: Delivery to the children of the “student kit”.

Step 5: Nutritional survey to estimate the nutritional habits of the children. This was carried out through two survey steps, by providing the aforementioned questionnaire: (i) in November, to know the current eating habits of children; and (ii) in May, to evaluate the project efficacy.

An interactive website was created to help the children build powerful bones, encompassing “online” games to teach a correct lifestyle for having a strong and healthy skeleton. The website featured a description of the project and an educational section on bone science and metabolism, along with information about nutrition for correct skeletal development especially developed and amusing for school-aged children. Two different levels of access were made possible, one for the general public, and another for the participants in the project. Visiting this website, children could learn how to increase calcium and vitamin D intake through both diet and physical activity, in an easy, gamified and amusing way. In particular, the *game section* encompassed a game based on finding wrong and right behaviors of a cartoon character (Baby Bone) towards bone health, crosswords puzzle and quizzes about bone metabolism and good nutrition. The results of the total score obtained by each subjects were displayed and constantly updated on the homepage of the website.

A colorful “Mister Bone Calendar” allowed the children to track their calcium intake and physical activity by using thematic stickers, with active participation in daily progress. Each month, a graphic element with Mister Bone^®^ as the first actor provided important advice about nutrition and bone health.

The nutritional education project was carried out for one full school year, from November 2013 to May 2014, in the 4th and 5th grades of two selected primary schools in Florence. The first step was taken in November and was dedicated to nutritional education with particular emphasis on dairy nutrition and the importance of the adequate intake of calcium and other nutrients necessary for bone health. A 60-min seminar and discussion with the children was performed by dedicated nutritionists, focusing on optimal dietary calcium intake throughout childhood and adolescence, and its significance and impact on PBM. The presentation included: the basic concepts of physiology of bone; the importance of nutrition and physical activity for bone health; strategies for increasing calcium and vitamin D in the diet; appropriate calcium-rich food sources; the relevance of having a regular breakfast; the importance of exposure to sunshine; and the risk factors for PBM accrual. Slides included pictures of foods rich in calcium and vitamin D, and the Recommended Dietary Allowance (RDA) requirements for calcium and vitamin D for children. The slide show was followed by an interactive group discussion regarding doubts and questions.

**Figure 1:**
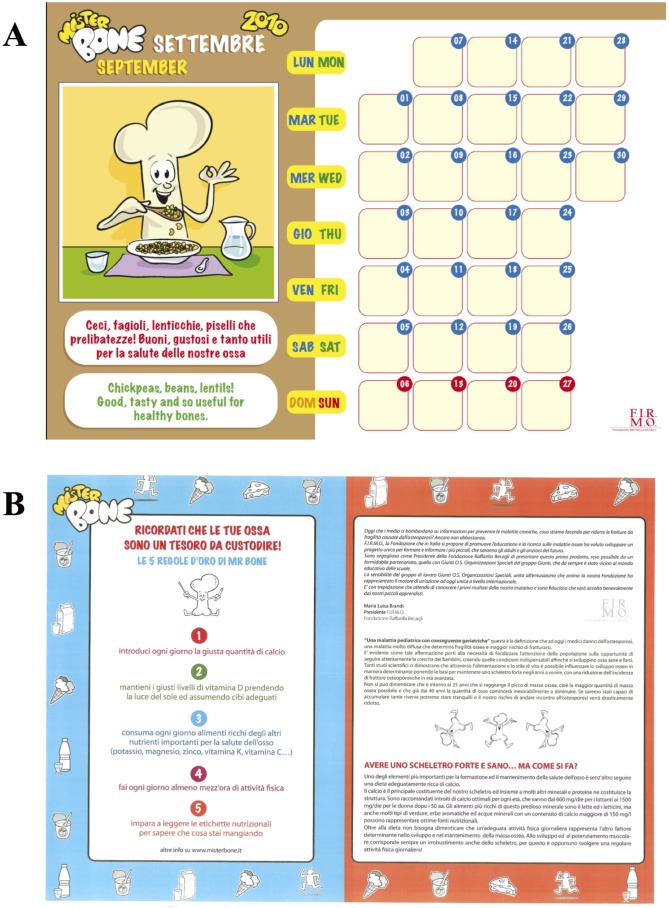
Educational paper support realized for the project: (**A**) calendar; and (**B**) informative leaflet.

Children received a “student kit” which included the “Mister Bone Calendar”, an informative brochure describing the contents of the initiative, and a compact disc with all the project contents, including the games created for the project (Figure 1).

### 2.4. Nutrients Analysis

A database was realized on the basis of the supplied questionnaires through a software program that included the Tables of Food Composition compiled by the Italian National Institute of Nutrition (WinFood 2.7-MediMatica). The nutritionists who performed the interview calculated the food and nutrient intake, using the questionnaires completed during the two-step survey.

### 2.5. Statistical Analysis

Data from 156 subjects (the ones who were present at the evaluation after 7 months) were analyzed to compare attitudes and consumption frequency before and after the educational program, with particular interest in the intake of total energy (Kcal/day), calcium (mg/day), vitamin D (µg/day), protein (mg/day), lipids and carbohydrates (mg/day).

Additional calculated and analyzed variables (*i.e.*, micronutrients) were: starch, vitamin A, niacin, riboflavin, thiamin, vitamin B6, folic acid, purine, potassium, zinc, phosphorus, iron, energy, vitamin C, vitamin D, vitamin E, oxalic acid, selenium, sodium, fiber, magnesium, and copper.

An analysis of the amount of specific foods consumed that are important for bone health, such as milk, fresh and hard cheeses, and vegetables, was also carried out.

A preliminary statistical analysis of baseline data obtained from the two schools was performed by applying a paired-sample Student’s *t*-test.

Each nutrient was analyzed with a repeated measures ANOVA with a 2-levels within-factor (Educational program: baseline and follow-up) × a 2-levels between-factor (Gender: males and females).

Results were considered as significant when the *p*-value was lower than 0.05.

## 3. Results

Table 1 shows the distribution of the group of children interviewed at baseline (176 children) and the group of children who completed the follow-up (156 children), according to gender and class of age.

A preliminary statistical analysis was carried out to rule out any difference in the distribution of the average intake of nutrients between the two primary schools, before and after the nutritional education. No statistically significant differences were observed. This allowed the pooling of results from the two schools (Table A1).

**Table 1:** Composition of the study group: all children at baseline (panel A), children who completed follow-up (panel B).

Age (years)	Male	Female	Row Total
**A**			
9–10	44 (52.38%)	50 (54.34%)	94 (53.40%)
10–11	40 (47.61%)	42 (45.65%)	82 (46.59%)
Column total	84 (47.72%)	92 (52.27%)	176 (100.00%)
**B**			
9–10	43 (58.10%)	41 (50.00%)	84 (53.85%)
10–11	31 (41.90%)	41 (50.00%)	72 (46.15%)
Column total	74 (47.43%)	82 (52.57%)	156 (100.00%)

As expected, the male intake of calories was statistically higher than the female intake (1691 ± 290 kcal/day in boys and 1613 ± 256 in girls, F_(1,154)_ = 4.94, *p* < 0.05). Mean calcium intake at baseline was 865 ± 190 mg/day in the female group and 890 ± 200 mg/day in the male group, far below the LARN (Level Assumption Recommended Nutrients) levels recommended by the Italian Nutrition Society (110–1300 mg/day) (Table 2).

The mean caloric intake did not change between baseline and follow-up in both sexes (*p* = 0.344). In addition, no interaction effect between sex and the educational program was observed (*p* = 0.890) (Table 2).

The intake of many macro and micronutrients increased significantly from the baseline levels to follow-up levels, ensuing from the educational program (Table 2 and Table A2). As far as mineral metabolism is concerned, a significant increase in the intake of calcium (F_(1,154)_ = 106.09, *p* < 0.001), vitamin D (F_(1,154)_ = 19.25, *p* < 0.001), proteins (F_(1,154)_ = 64.17, *p* < 0.001), phosphate (F_(1,154)_ = 78.24, *p* < 0.001), magnesium (F_(1,154)_ = 86.22, *p* < 0.001), water (F_(1,154)_ = 101.47, *p* < 0.001), saturated fats (F_(1,154)_ = 6.02, *p* < 0.05), insoluble fiber (F_(1,154)_ = 26.57, *p* < 0.001) and soluble fiber (F_(1,154)_ = 13.60, *p* < 0.001) was observed. The intake in males was statistically higher compared to the intake in females only in the case of calcium (F_(1,154)_ = 4.13, *p* < 0.05) and saturated fats (F_(1,154)_ = 4.06, *p* < 0.05). No interaction effects were observed for any nutrients under scrutiny.

In particular, mean calcium intake rose from 882 ± 184 mg/day to 1078 ± 233 mg/day, and vitamin D intake increased from 3.4 μg/day to 4.1 μg/day as a result of the educational program and the increased daily consumption of foods important for bone health, such as milk, fresh and hard cheeses, and vegetables (Table 3). Children who started to drink milk were about 4% of total sample, and those who started to consume fresh and hard cheeses were 7%. The amount of milk drunk by children increased from 200 mL/die (1 glass) to 270 mL/die (1 cup) both in boys and girls. The intake of fresh and hard cheeses increased by about 30 g per week. The percentage of children eating cheese increased from 84% to 91%, and the percentage of those eating vegetables varied from 89% to 96%, and milk consumption from 92% to 96%. The proportion of children who used grated Parmesan cheese on pasta increased by 28% during the educational program.

**Table 2:** Mean values of energy intake, and the intake of water, and different macro and micronutrients in males (M), females (F) and the whole population (156) at baseline and follow-up after nutritional intervention.

Variables		Baseline Mean	Baseline SD	Follow-up Mean	Follow-up SD	*p*
Energy intake (Kcal/day)	M	1691	±291	1722	±337	*p* = NS
	F	1613	±276	1636	±252	
	Tot	1652	±285	1679	±297	
Calcium (mg/day)	M	898	±190	1118	±238	*p* < 0.001
	F	865	±179	1039	±224	
	Tot	882	±184	1078	±233	
Vitamin D (μg/day)	M	3.453	±1.6	4.081	±2.2	*p* < 0.001
	F	3.488	±1.4	4.197	±2.0	
	Tot	3.471	±1.5	4.129	±2.1	
Phosphate (mg/day)	M	1136	±206	1303	±244	*p* < 0.001
	F	1086	±170	1245	±212	
	Tot	1110	±190	1272	±228	
Magnesium (mg/day)	M	140.2	±49	178.9	±48	*p* < 0.001
	F	142.6	±38	176.6	±45	
	Tot	141.4	±43	177.7	±46	
Water (mL/day)	M	609.3	±160	750.3	±180	*p* < 0.001
	F	592.1	±138	738.1	±181	
	Tot	600.3	±149	743.9	±180	
Proteins (g/day)	M	64.24	±12	72.52	±14	*p* < 0.001
	F	60.95	±10	69.52	±12	
	Tot	62.51	±11	79.94	±13	
Saturated fats (g/day)	M	20.29	±4.0	21.90	±4.6	*p* < 0.05
	F	19.77	±4.2	20.21	±4.3	
	Tot	20.02	±4.1	21.01	±4.5	
Monounsaturated fats (g/day)	M	29.98	±7.0	27.68	±9.8	*p* < 0.05
	F	30.42	±7.4	28.47	±11.0	
	Tot	30.21	±7.2	28.09	±10.4	
Polyunsaturated fats (g/day)	M	6.51	±1.4	6.42	±1.7	*p* = NS
	F	6.33	±1.4	6.40	±1.5	
	Tot	6.42	±1.4	6.41	±1.6	
Carbohydrates (g/day)	M	227.11	±49.93	228.70	±57.66	*p* = NS
	F	210.26	±50.52	209.82	±42.59	
	Tot	218.26	±50.78	218.79	±51.01	

*p* values refer only to the main effect of the intervention independently of sex; NS = non-significant; Tot: Total.

**Table 3:** Intake of milk, fresh and hard cheese, and vegetables before and after the educational program.

		Milk (mL/Day)		Fresh and Hard Cheeses (g/Week)		Vegetables (g/Day)	
		Mean	SD	Mean	SD	Mean	SD
**Girls**	Pre-educational intervention	198	37	49	29	158	88
	Post-educational intervention	265 **	71	59 *	44	204 **	91
**Boys**	Pre-educational intervention	204	39	54	32	164	107
	Post-educational intervention	280 **	63	59 *	34	217 **	98

* *p* not significant and ** *p* < 0.05 versus baseline/pre-educational values.

The daily intake of fresh vegetables increased by about 50 g/day (from 160 ± 97 to 210 ± 94 g/day) during the educational program.

Polyunsaturated fats did not change between baseline and follow-up (*p* = 0.954) and there were no sex-related differences (*p* = 0.603). Monounsaturated fats decreased between baseline and follow-up measurements (F_(1,154)_ = 5.66, *p* < 0.05) and there were no sex-related differences (*p* = 0.586) (Table 2).

The intake of other micronutrients, such as potassium, zinc, iron, vitamin C, magnesium, selenium, copper, niacin, thiamine, and riboflavin increased significantly (*p* > 0.05) following application of the educational program (Table A2).

Table 4 shows the changes in the composition of the diet before and after the educational program, in terms of percentage of macronutrients. The percentage of proteins increased by two points, from 14.5% to 16.5%, while for both carbohydrates and lipids there was a decrease of one percentage point.

**Table 4:** Diet composition as macronutrients percentages, before and after the educational program.

	Pre-Educational	Post-Educational
Proteins	14.5%	16.5% *
Lipids	34%	33% *
Carbohydrates	51.5%	50.5% *

* *p* not significant *versus* baseline/pre-educational values.

## 4. Discussion

The achievement of optimal PBM through accrual of mineral mass during infancy, childhood and adolescence is an important determinant of bone health to prevent osteoporosis in adulthood. Although genetic factors are prevalent elements in the variance of PBM, some other factors, such as diet, are amenable to intervention during infancy, childhood and adolescence. Increasing daily calcium intake with dairy products, correcting vitamin D insufficiency, increasing fruit and vegetable intake, and daily physical activity, all contribute to the maintenance and improvement of skeletal health in order to prevent osteoporosis and the associated risk of fracture in childhood, adolescence and adulthood [5,12,40,41,42]. Nonetheless, dairy product consumption tends to decrease after the school-age period [43]. Therefore, there is a need to develop and implement effective programs and policies that will result in children and adolescents adopting healthier diets to support bone strength.

Schools are a crucial social environment for children and adolescents, and many attempts have been made to utilize this environment to promote healthy behavior in youth, including good eating habits [44].

The Mister Bone study has addressed these issues by applying a unique seven-month educational program with a gamified approach to schoolchildren, in order to improve their eating behavior, with particular respect towards the nutrients crucial for bone health.

The age of 9–11 years was chosen for two main reasons: (1) it is critical for achieving the optimal PBM required to prevent osteoporosis and its consequences; and (2) children at this age are fully competent in acquiring proper information and cooperating successfully in the reliable completion of questionnaires [45,46].

The results obtained from this study show that this nutrition educational program was effective in modifying the nutrition of children by increasing the intake of nutrients important for bone health intake, such as calcium, phosphate, and vitamin D, as well as polyvalent foods, such as dairy products (e.g., milk and cheese), which contain many essential nutrients, such as protein and calcium, with effects on bone health exceeding the sum of the single elements.

In particular, a significant increase in calcium intake before and after the educational program was demonstrated. This is a major achievement, considering that in the Italian population mean daily calcium consumption is about 76% of the average recommendation [23]. In line with previous results, in the Mister Bone study, the calcium intake of the children before the educational intervention was about 75% of the recommended Italian LARN levels [25]. At the end of the study period, although not yet optimal, the calcium intake significantly rose to 1039 mg/day in girls and 1118 mg/day in boys, representing up to 90% of the recommended intake.

Vitamin D is a key hormone for the regulation of bone growth and mineralization throughout life. Many studies show that deficiency or inadequacy of vitamin D in children and adolescents living in several regions of the world [47], with consequent decrease in bone mineralization, especially during periods of accelerated bone growth, lead to long-term detrimental bone effects. Although adequate sun exposure is recommended, optimal vitamin D intake through nutrition and/or use of supplements during infancy, childhood, and adolescence is equally important. Although the results achieved in this study are still far from attaining the levels recommended by LARN for vitamin D intake (15 µg/day), the educational program led to a small but significant increase in its dietary intake in schoolchildren. The consumption of fortified foods (e.g., milk, juice, and cereals enriched in vitamin D), although not specifically quantitated, might be the cause of these changes, since the program endorsed their use.

It is worth noting that all changes took place without variation in the total energy intake, and independently of the sex of the child.

Evidence suggests a positive association between the amount of ingested proteins and bone mass gain in healthy children and adolescents of both sexes, with this association being particularly relevant in pre-pubertal children [48]. As a result of the intervention, protein intake increased in both sexes. This is probably due to a major shift in the quality of food consumed. Indeed, our data showed that children increased their consumption of dairy products or, in some cases, began to consume them. An increase in the consumption of fat-free dairies and whole-grains could be inferred since the total energy and carbohydrates intake did not change, with an only modest rise in consumption of saturated, monosaturated, and polyunsaturated fatty acids. Unfortunately, the intake of fat-free foods and whole cereals was not precisely quantified in the administered questionnaire and this may represent a limitation of the study.

The success of our educational program is highlighted by several observations on small but fundamental changes of dietary habits, with increased consumption of milk, fresh and hard cheeses, and vegetables. These results are important because the consumption of dairy products at a young age may influence dietary habits later in life. The increase in the consumption of a cheese like Parmesan, which can offer a good calcium intake option even for intolerant people, given the absence of lactose, is very important.

Daily vegetable intake is very important for children, not only for vitamin and mineral intake, but also for their contribution in preventing renal calcium loss [49]. Similar to what happened with milk and cheese, the daily intake of vegetables increased, probably as a consequence of the educational program.

This all took place at an age (9–11 years) that is crucial, since the consumption of dairy products tends to decrease further during adolescence, with a consequent increase in the risk of fracture before puberty (a 2.6-fold higher risk has been reported), and possibly later in life [50,51].

So far, only one other pilot study applying a similar educational program for a small group of schoolchildren (12 subjects) with a similar aim (increasing calcium intake) has been found in the literature [52]. With respect to this initial experience, the Mister Bone nutritional intervention has been demonstrated to be effective in a significantly larger group of schoolchildren and, given its characteristics, can easily be scalable up to an even larger comparable population.

We acknowledge some limitations of this study do exist, such as the lack of a control group, the relative small number of subjects evaluated and enrolled in the program, the lack of anthropometric measurements and blood tests to assess vitamin D status and other metabolic parameters. Nonetheless, we also underline that this progressive program, realized through lessons, leaflets, and games, is unique, also from an educational point of view, developing and supporting automated positive behaviors according to the *nudge* *theory* [53]. Exploiting the psychological distinction between intuitive and deliberate reasoning processes [54,55], this theory employs positive reinforcements, and indirect, non-forced suggestions, in order to more easily influence the decision making of groups and individuals. This is critical, since long-term changes in nutritional habits are required for the maintenance and promotion of good health. In this context, the gamified approach and self-monitoring are very effective in promoting and sustaining good automated behaviors, especially in children and adolescents [56]. As far as empowerment of children for good choices is concerned, the intervention we propose can yield an added value with respect to other interventions obtained by food fortification or special school programs, which are imposed and not truly autonomously chosen by individuals.

For all the above-mentioned reasons, the Mister Bone educational program may be considered a new instrument of primary prevention to improve eating behaviors, increasing calcium and vitamin D intakes in larger groups of healthy schoolchildren. The findings ensuing from this pilot study may encourage school policies to promote educational strategies for the promotion of skeletal health.

## 5. Conclusions

An original educational program of primary prevention carried out in Italian primary schools and specifically designed for children aged 9–10 years, has been demonstrated to be effective in improving good nutritional habits in particular aimed to sustain bone health. This has happened in an age range, which is crucial for bone accrual and prevention of osteoporosis later in life.

This instrument can be further exploited and tested in different and larger backgrounds in order to increase knowledge and empower children about good choices for maintaining or improving their skeletal strength.

## Appendix

**Table A1:** Comparison between the two schools at baseline: *T*-test results.

Variables	*t*-Value	*p*
calcium	–0.52	0.6069
protein	–0.08	0.9398
lipids	0.68	0.4977
carbohydrates	0.77	0.441
starch	1.04	0.2979
vitamin A	–0.91	0.3654
niacin	–0.09	0.9295
riboflavin	–0.98	0.329
thiamin	–0.60	0.5488
vitamin B6	–0.88	0.3793
folic acid	–0.44	0.6598
purine	–1.82	0.0702
potassium	–0.26	0.7965
zinc	0.13	0.8975
phosphorus	0.11	0.9129
iron	–1.34	0.1823
energy	0.73	0.4635
vitamin C	–0.42	0.6738
vitamin D	–2.13	0.0344
vitamin E	1.23	0.2187
oxalic acid	–1.29	0.1987
selenium	–0.62	0.5335
sodium	0.59	0.5555
fibers	0.40	0.6882
magnesium	–0.20	0.8436
copper	–0.00	0.9996

**Table A2:** Mean values of the intake of additional macro and micronutrients, which significantly increased in the whole population (156) at baseline and follow-up after nutritional intervention (*p* < 0.05).

Variables	Baseline	Follow-up
Fibers (g/day)	15.3	16.3
Potassium (mg/day)	1843.2	2218.6
Iron (mg/day)	9.9	11.2
Zinc (mg/day)	8.3	9.5
folic acid (mcg/day)	273.8	327.5
Vitamin B1 (mg/day)	1.1	1.3
Vitamin B2 (mg/day)	1.4	1.8
Vitamin B3 (mg/day)	9.6	11.3
Vitamin B6 (mg/day)	1.3	1.5
Vitamin A (mcg/day)	759.1	905.8
vitamin C (mg/day)	86.1	111.6
Selenium (mcg/day)	31.1	36.1
Copper (mg/day)	1.0	1.3
Purines (mg/day)	47.1	56.0
